# *Mycoplasma pneumoniae* outbreak, Southeastern Finland, 2017–2018: molecular epidemiology and laboratory diagnostic lessons

**DOI:** 10.1007/s10096-019-03619-7

**Published:** 2019-07-01

**Authors:** Satu Kurkela, Mirja Puolakkainen, Kati Hokynar, Tea Nieminen, Harri Saxen, Laura Mannonen, Risto Pietikäinen

**Affiliations:** 1 Department of Virology, University of Helsinki and Helsinki University Hospital, Helsinki, Finland; 2 Children’s Hospital, University of Helsinki and Helsinki University Hospital, Helsinki, Finland; 3 Kymenlaakso Central Hospital, Kotka, Finland

**Keywords:** *Mycoplasma pneumoniae*, Pneumonia, Respiratory tract infections, Disease outbreaks, Nucleic acid amplification tests, Bacterial antibodies

## Abstract

This study characterizes a large *Mycoplasma pneumoniae* outbreak observed in Kymenlaakso in Southeastern Finland during August 2017–January 2018. The first part of the investigation included 327 patients, who sought healthcare consultation at local GPs or hospitals due to clinical symptoms, and were tested for *M. pneumoniae* antibodies (Patient cohort). The second part of the investigation, conducted approximately 4 weeks after the peak of the outbreak, consisted of school screening of pupils (*N* = 239) in three different school buildings by PCR on respiratory specimens and questionnaires (Screening cohort). PCR positive respiratory specimens were subsequently utilized for molecular typing. The outbreak peaked in late October 2017. Of the Patient cohort, 9/106 (8.5%) respiratory specimens were PCR positive. In contrast, 3/182 (1.6%) of the Screening cohort were PCR positive. Asymptomatic carriage was observed. Multiple-locus variable-number tandem-repeat analysis (MLVA) identified two distinct MLVA types. All typed *M. pneumoniae* strains belonged to P1 type 1. No mutations leading to macrolide resistance were observed. In total, 61/327 (19%) of the Patient cohort had a serological indication of recent infection. The IgM test reactivity at the time of a negative PCR test result varied from a completely non-reactive value up to very strong reactivity, highlighting the difficulty in a single specimen serodiagnosis.

## Introduction

*Mycoplasma pneumoniae* is a common causative agent of community-acquired pneumonias, and together with *Chlamydia pneumoniae* cause atypical pneumonias. The highest incidence rates for mycoplasma pneumonia is seen in children and young adults, and it is typically characterized by a slowly progressing onset, followed by a persisting cough [1]. While mycoplasma pneumonias are treated with antibiotics, the majority of cases present with a milder respiratory disease of self-limiting nature which does not require antimicrobial treatment.

For *M. pneumoniae* laboratory diagnostics, both antibody detection and nucleic acid amplification (typically PCR) are used. They both have their advantages and limitations in terms of clinical context and time elapsed from disease onset. While their analytical performance can be optimized, the interpretation can be challenging due to e.g. long-term IgM persistence after infection, sometimes reduced IgM reaction in reinfections, and asymptomatic carriage of *M. pneumoniae* in the respiratory tract [2].

The aim of this study was to characterize a *M. pneumoniae* outbreak observed in Kymenlaakso in Southeastern Finland during August 2017–January 2018 by clinical questionnaires, serology, and molecular typing and to inform the use of laboratory diagnostics in the management of *M. pneumoniae* infections.

## Materials and methods

### Study design

Two separate investigations were conducted (Table 1). The Patient cohort included all patients, who were tested for *M. pneumoniae* antibodies between 1 August 2017 and 31 January 2018 due to healthcare consultation and a clinically suspected *M. pneumoniae* infection at local GPs or hospitals in the Kymenlaakso region. Two or more sera were available from 67/327 (20%) of the cases.

**Table 1:** Case descriptions, diagnostic findings, and clinical characteristics

	Patient cohort*	Screening cohort*	Overall*
Cases, *N*	327	239	535
Males, *N* (%)	155 (47.4)	112 (46.9)	249 (46.5)
Age (years), mean	37	13	28
Age (years), range	(0, 89)	(7, 57)	(0, 89)
Cases with respiratory specimen(s) retrieved, *N*	106	182	262
Cases with *M. pneumoniae* PCR positive result from respiratory specimen, *N* (%)	9 (8.5)	3 (1.6)	11 (4.2)
Case with serum specimen(s) retrieved for *M. pneumoniae* antibody testing, *N*	327	31	327
Cases with seroconversion, *N* (%)	12 (3.7)	2 (6.5)	12 (3.7)
Cases with either IgM > 3.0 S/CO AND IgG > 45 EIU; or more than a twofold IgG rise, *N* (%)	49 (15.0)	20 (64.5)	49 (15.0)
Questionnaire, *N*	29	208	208
Fever, yes/no (%)	25/2 (92.6)	123/74 (62.4)	123/74 (62.4)
Cough, yes/no (%)	26/0 (100)	120/33 (78.4)	120/33 (61.4)
Rhinorrhoea, yes/no (%)	15/6 (71.4)	107/40 (72.9)	107/40 (72.8)
Antibiotics, yes/no (%)	20/5 (80.0)	43/98 (30.5)	43/98 (30.5)
Chest radiograph, yes/no (%)	193/134 (59.0)	27/212 (11.3)	193/134 (59.0)
Pneumonia in chest radiograph, yes/no (%)	104/90 (53.6)	20/7 (74.1)	104/90 (53.6)

*31 cases were included in both the Patient cohort and the Screening cohort

The Screening cohort consisted of screening of 236 pupils (aged 7–22 years) and three staff members from three school buildings in the municipality of Virolahti in Kymenlaakso (between 27 November and 1 December 2017), which were selected due to reports of unusually frequent respiratory infections and absentees. Nasopharyngeal swabs and questionnaires were collected, when possible (Table 1). PCR positive swabs were utilized for molecular typing.

Altogether 535 cases were included in the study: the Patient cohort consisted of 327 cases; the Screening cohort consisted of 239 cases, and 31 cases were included in both cohorts.

### Serological testing for *M. pneumoniae*

Serology was conducted as routine testing at the Helsinki University Hospital Laboratory with Labsystems *M. pneumoniae* IgM and IgG EIA by following the manufacturer’s instructions (Labsystems Diagnostics, Vantaa, Finland). The dynamic range of the IgG EIA was set between 45 and 400 EIU, and test results below 45 EIU were considered as negative. To increase specificity, the cut-off for positive IgM reaction was considerably hightened from the manufacturer’s 1.2 S/CO to 3.0 S/CO for this investigation.

### Detection of *M. pneumoniae*

DNA was extracted using MagNA Pure LC Total Nucleic Acid Isolation Kit or MagNA Pure DNA/Viral NA SV 2.0 Kit (Roche Diagnostics GmbH, Mannheim, Germany). In detail, 300 μl of the specimen was lysed with 300 μl of MagNA Pure Lysis/Binding Buffer. Five hundred microliters or 450 μl of the lysate was extracted with the MagNA Pure LC or the MagNA Pure 96-instrument, respectively. A multiplex real-time PCR assay for simultaneous detection of *M. pneumoniae*, *Chlamydia pneumoniae*, and mutations most commonly associated with macrolide resistance in *M. pneumoniae* developed by us [3] was used. This assay amplifies part of the *M. pneumoniae* hypothetical protein C985_0367 gene. The macrolide resistance-associated mutations in *M. pneumoniae* strains were identified by amplification of the 23S rRNA gene followed by a dissociation curve analysis, which detects mutations at sequence positions 2063G/C and 2064G/C.

### P1 gene typing

The type of P1 adhesin protein gene was determined as described earlier [4] using a multiplex real-time PCR assay for detection of defined P1 types, based on the presence of short type-specific indels in the *M. pneumoniae* genomic sequence. DNA from a reference strain M129 (ZeptoMetrics Corporation, Franklin, MA) and from an earlier clinical isolate were used as controls (type 1 and type 2, respectively).

### Multi-locus variable-number tandem-repeat analysis (MLVA) typing

Five selected loci containing tandem repeats (Mpn1 and 13-16) were amplified as described earlier [5]. The PCR products were sequenced, and the VNTR number of each locus was determined using tandem repeat finder software (http://tandem.bu.edu/trf/trf.html) [6] with settings suggested by Chalker et al. [7]. DNA from a reference strain M129 was used as control (type P).

### Questionnaire

A standardized questionnaire was filled in by the pupils or their guardians in the school screening investigation. Data included demographic information, illness characteristics, and use of antibiotics.

## Results

**Fig. 1:**
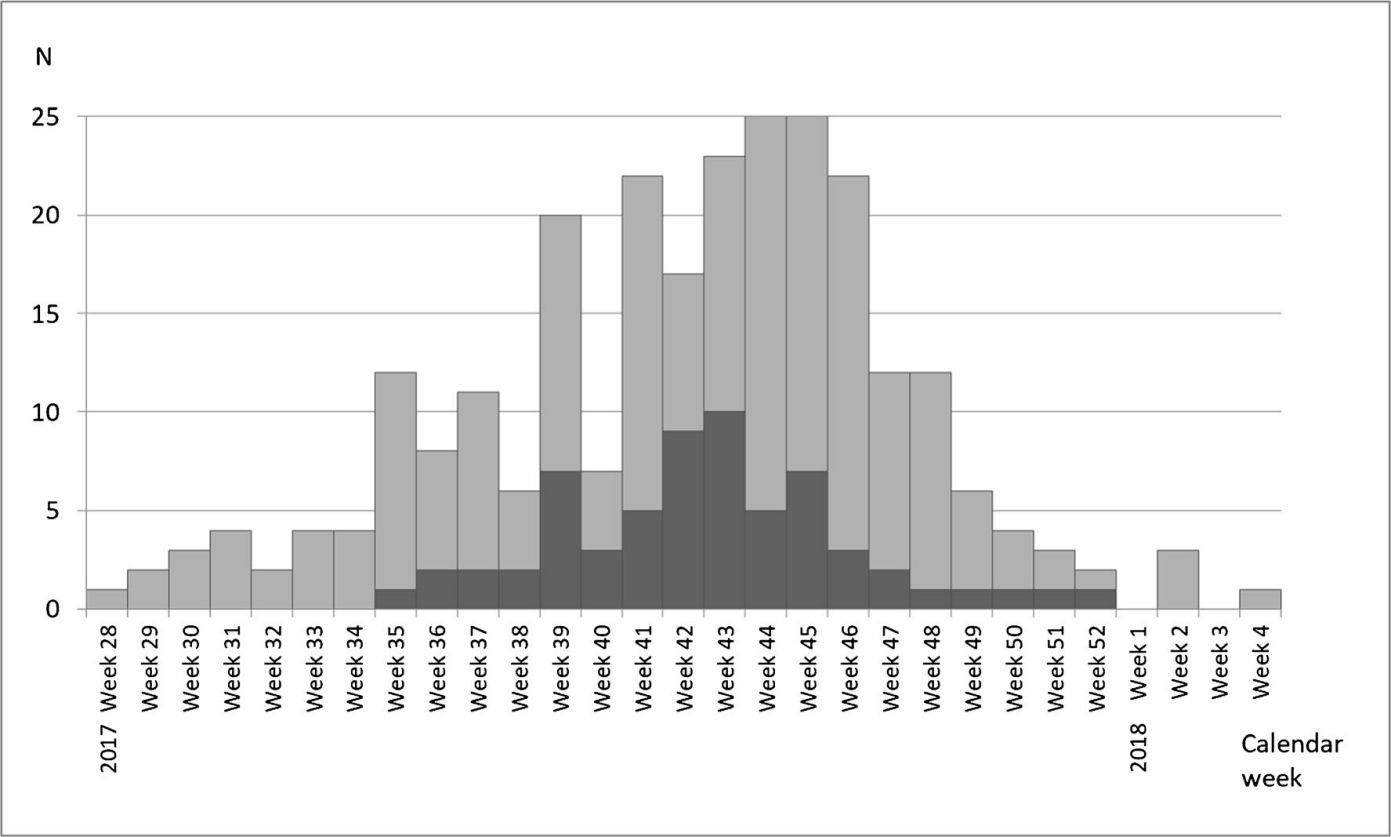
Epidemic curve. Only those cases (*N* = 261) of whom the onset of illness was known are depicted in the curve (calendar weeks 28–52 of 2017 and 1–4 of 2018). Black cases showed laboratory evidence of acute or recent *M. pneumoniae* infection (a PCR positive test result in the respiratory specimen, or a seroconversion or more than a twofold rise in IgG EIU between paired serum samples, or a serology with IgM > 3.0 S/CO and IgG > 45 EIU). Light grey cases were symptomatic without definitive laboratory evidence of *M. pneumoniae* infection

The 2017 *M. pneumoniae* outbreak in the Kymenlaakso region, Finland began in August and peaked during late October and early November (Fig. 1). Prior to week 28, there were only sporadic cases diagnosed in Kymenlaakso.

Twelve cases showed a seroconversion, and three cases showed a more than a twofold increase in IgG in paired sera. Of these, a swab was available from 11/15 cases, of whom 5/11 were PCR positive. In addition, there were six PCR positive cases of whom there was no serological evidence due to lack or timing of serum specimens. One PCR positive pupil had no symptoms. A total of 123/197 (62%) of those who returned a questionnaire reported having fever (Table 1). The negative predictive value for a PCR negative result among those not reporting fever was 34%.

**Fig. 2:**
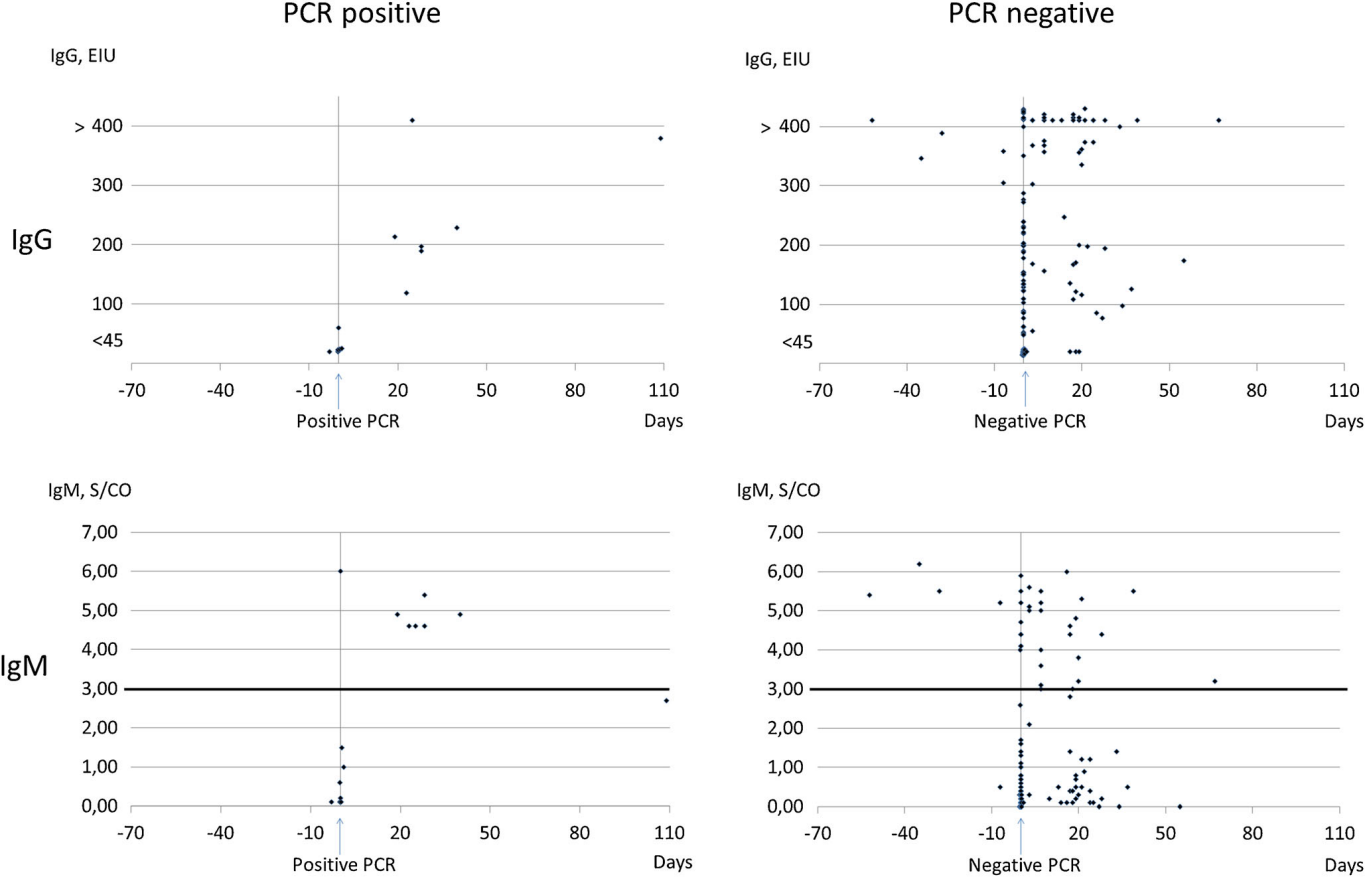
*M. pneumoniae* IgG and IgM antibody test results in relation to timing and result of PCR testing. For one of the PCR positive cases, no serum was available for antibody testing

**Fig. 3:**
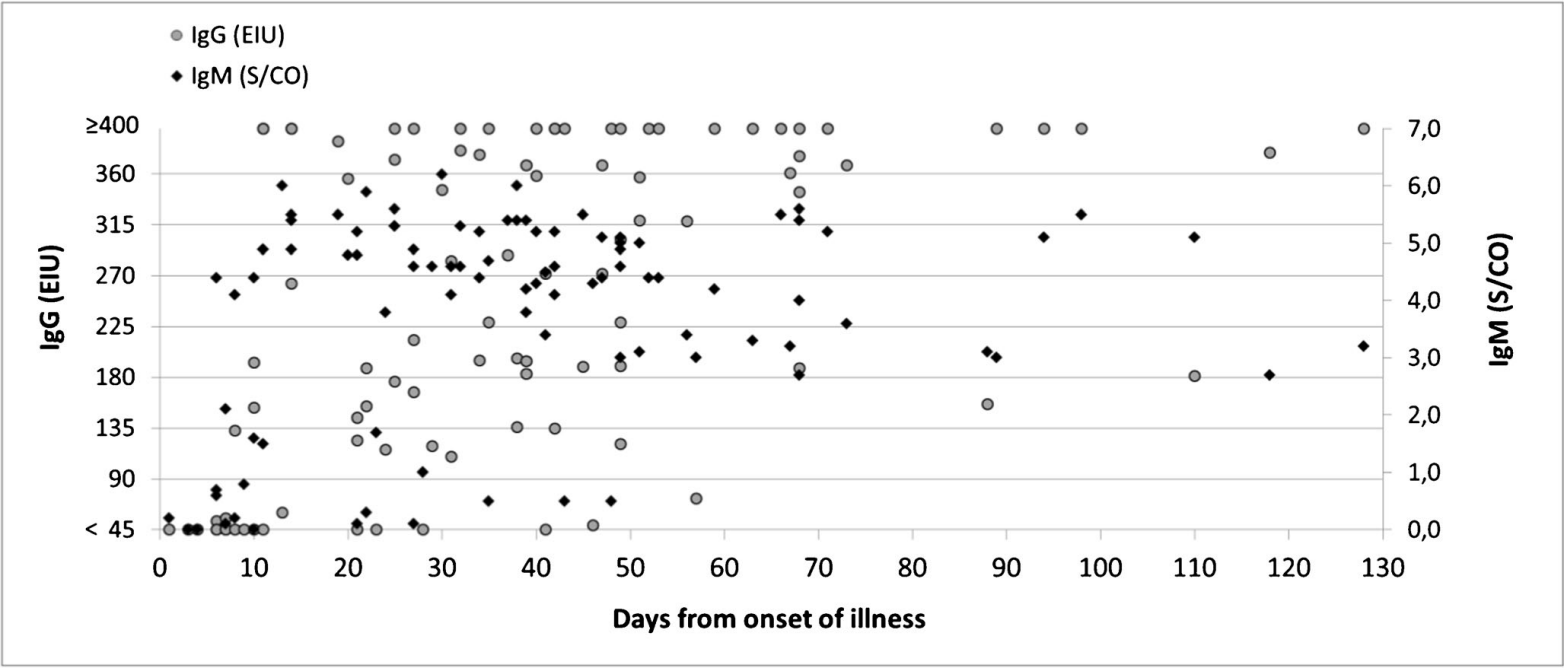
*M. pneumoniae* IgG and IgM antibody test results in relation to onset of illness among the cases (63 cases, 95 specimens) who had laboratory evidence of acute or recent *M. pneumoniae* infection (a PCR positive test result in the respiratory specimen, or a seroconversion or more than a twofold rise in IgG EIU between paired serum samples, or a serology with IgM > 3.0 S/CO and IgG > 45 EIU)

The IgM test values (S/CO) at the time of a negative PCR test result varied from a completely non-reactive value (< 1.2 S/CO) up to very strong reactivity (6.0 S/CO) (Fig. 2). IgM and IgG response was typically observed during the second week post onset of illness (Fig. 3). IgM persisted for several months, with S/CO values peaking approximately 4 weeks post onset, and declining by the time of 8 weeks post onset.

*M. pneumoniae* DNA was amplified in 12 specimens. Eleven of these were typed according to the *M. pneumoniae* P1 protein, and all were of type 1. Altogether, 10 *M. pneumoniae* strains were classified into 5-locus MLVA types (Mpn1, Mpn13-16). When the most discriminatory, but unstable Mpn1 locus was excluded, nine strains were of type 4-5-7-2 and one of type 3-5-7-2. The most common macrolide resistance-associated mutations (in positions 2063G/C and 2064G/C of the 23S rRNA sequence) were not detected in the specimens.

## Discussion

Epidemics of *M. pneumoniae* infection have occurred at 3–7-year intervals in Scandinavian countries and elsewhere [8]. In Finland, a large *M. pneumoniae* epidemic was observed serologically in 2010–2011 [9], but the circulating strains were not characterized.

In line with earlier reports [10], the most variable locus of M. pneumoniae was Mpn1 (repeat numbers ranging from three to six) in the Kymenlaakso 2017 outbreak. When the Mpn1 locus was excluded, MLVA type 4-5-7-2 was the most common, and in agreement with studies from other regions of the world. Two MLVA types were identified suggesting the presence of different MLVA types during the same outbreak. All strains were of P1 type 1. Although our earlier study showed that 9.5% of Finnish *M. pneumoniae* strains had mutations conferring resistance to macrolides [3], no such mutations were observed among these strains.

One of the PCR positive school pupils reported no symptoms in the questionnaire, reflecting an asymptomatic carriage [2]. The low PCR positivity rate (1.6%) in the respiratory specimens observed in the school screening may be due to the late timing of specimen collection (week 48) in relation to the peak of the outbreak (weeks 43−45) (Fig. 1) or due to prior use of antibiotics. While in some cases *M. pneumoniae* can be detected several months after infection, the carriage typically clears within the first month post infection [2], and our study is in line with this observation.

The best IgM antibody tests for *M. pneumoniae* have an approximately 70–80% sensitivity and 90% specificity [11]. The very variable IgM test values with a simultaneous negative PCR test result in a respiratory specimen (Fig. 2) as well as IgM persistence (Fig. 3); both showcase the difficulty in the clinical interpretation of an IgM reactive test result in a single specimen. The relatively low rate of serodiagnoses (19%) in the Patient cohort may be due to suboptimal timing of specimen collection and lack of paired serum specimens.

Combination of serology and PCR has been suggested to increase diagnostic precision [12], but such an approach may not be feasible in clinical practice. The use of *M. pneumoniae* laboratory diagnostics should be primarily limited for cases with the intention to treat with antimicrobials, or for surveillance purposes. In such settings, laboratory data combined with clinical and/or epidemiological data will provide the necessary information for decision making.

## References

[1] Waites KB, Xiao L, Liu Y, Balish MF, Atkinson TP (2017). Mycoplasma pneumoniae from the respiratory tract and beyond. Clin Microbiol Rev.

[2] Spuesens EB, Fraaij PL, Visser EG, Hoogenboezem T, Hop WC, van Adrichem LN (2013). Carriage of *Mycoplasma pneumoniae* in the upper respiratory tract of symptomatic and asymptomatic children: an observational study. PLoS Med.

[3] Nummi M, Mannonen L, Puolakkainen M (2015). Development of a multiplex real-time PCR assay for detection of mycoplasma pneumoniae, chlamydia pneumoniae and mutations associated with macrolide resistance in mycoplasma pneumoniae from respiratory clinical specimens. Springerplus.

[4] Wolff BJ, Benitez AJ, Desai HP, Morrison SS, Diaz MH, Winchell JM (2017). Development of a multiplex taqMan real-time PCR assay for typing of *Mycoplasma pneumoniae* based on type-specific indels identified through whole genome sequencing. Diagn Microbiol Infect Dis.

[5] Dégrange S, Cazanave C, Charron A, Renaudin H, Bébéar C, Bébéar CM (2009). Development of multiple-locus variable-number tandem-repeat analysis for molecular typing of mycoplasma pneumoniae. J Clin Microbiol.

[6] Benson G (1999). Tandem repeats finder: a program to analyze DNA sequences. Nucleic Acids Res.

[7] Chalker VJ, Pereyre S, Dumke R, Winchell J, Khosla P, Sun H (2015). International mycoplasma pneumoniae typing study: interpretation of M. pneumoniae multilocus variable-number tandem-repeat analysis. New Microbes New Infect.

[8] Atkinson TP, Balish MF, Waites KB (2008). Epidemiology, clinical manifestations, pathogenesis and laboratory detection of mycoplasma pneumoniae infections. FEMS Microbiol Rev.

[9] Polkowska A, Harjunpää A, Toikkanen S, Lappalainen M, Vuento R, Vuorinen T et al (2012) Increased incidence of *Mycoplasma pneumoniae* infection in Finland, 2010-2011. Euro Surveill 17 10.2807/ese.17.05.20072-en 22321135

[10] Benitez AJ, Diaz MH, Wolff BJ, Pimentel G, Njenga MK, Estevez A (2012). Multilocus variable-number tandem-repeat analysis of *Mycoplasma pneumoniae* clinical isolates from 1962 to the present: a retrospective study. J Clin Microbiol.

[11] Beersma MF, Dirven K, van Dam AP, Templeton KE, Claas EC, Goossens H (2005). Evaluation of 12 commercial tests and the complement fixation test for *Mycoplasma pneumoniae*-specific immunoglobulin G (IgG) and IgM antibodies, with PCR used as the “gold standard”. J Clin Microbiol.

[12] Medjo B, Atanaskovic-Markovic M, Radic S, Nikolic D, Lukac M, Djukic S (2014). *Mycoplasma pneumoniae* as a causative agent of community-acquired pneumonia in children: clinical features and laboratory diagnosis. Ital J Pediatr.

